# Cardiac Autonomic Responses during Exercise and Post-exercise Recovery Using Heart Rate Variability and Systolic Time Intervals—A Review

**DOI:** 10.3389/fphys.2017.00301

**Published:** 2017-05-29

**Authors:** Scott Michael, Kenneth S. Graham, Glen M. Davis

**Affiliations:** ^1^Discipline of Exercise and Sports Science, Faculty of Health Sciences, University of SydneySydney, NSW, Australia; ^2^New South Wales Institute of SportSydney, NSW, Australia

**Keywords:** parasympathetic, HRV, vagal, sympathetic, pre-ejection period, reactivity, allostasis, challenge test

## Abstract

Cardiac parasympathetic activity may be non-invasively investigated using heart rate variability (HRV), although HRV is not widely accepted to reflect sympathetic activity. Instead, cardiac sympathetic activity may be investigated using systolic time intervals (STI), such as the pre-ejection period. Although these autonomic indices are typically measured during rest, the “reactivity hypothesis” suggests that investigating responses to a stressor (e.g., exercise) may be a valuable monitoring approach in clinical and high-performance settings. However, when interpreting these indices it is important to consider how the exercise dose itself (i.e., intensity, duration, and modality) may influence the response. Therefore, the purpose of this investigation was to review the literature regarding how the exercise dosage influences these autonomic indices during exercise and acute post-exercise recovery. There are substantial methodological variations throughout the literature regarding HRV responses to exercise, in terms of exercise protocols and HRV analysis techniques. Exercise intensity is the primary factor influencing HRV, with a greater intensity eliciting a lower HRV during exercise up to moderate-high intensity, with minimal change observed as intensity is increased further. Post-exercise, a greater preceding intensity is associated with a slower HRV recovery, although the dose-response remains unclear. A longer exercise duration has been reported to elicit a lower HRV only during low-moderate intensity and when accompanied by cardiovascular drift, while a small number of studies have reported conflicting results regarding whether a longer duration delays HRV recovery. “Modality” has been defined multiple ways, with limited evidence suggesting exercise of a greater muscle mass and/or energy expenditure may delay HRV recovery. STI responses during exercise and recovery have seldom been reported, although limited data suggests that intensity is a key determining factor. Concurrent monitoring of HRV and STI may be a valuable non-invasive approach to investigate autonomic stress reactivity; however, this integrative approach has not yet been applied with regards to exercise stressors.

## Introduction

Quantifying the fluctuations in R-wave to R-wave intervals (RRI), referred to as heart rate variability (HRV), has been considered a useful method by which to monitor autonomic activity, in particular cardiac parasympathetic modulation (Camm et al., 1996). Monitoring HRV responses to an “exercise challenge test” may provide useful insight into autonomic stress reactivity. This is consistent with the “reactivity hypothesis” (Krantz and Manuck, 1984; Heponiemi et al., 2007), which proposes that cardiovascular responses to a stressor may be predictive of certain diseases (Treiber et al., 2003; Lovallo, 2005; Phillips, 2011), as well as useful in monitoring the training status of high performance athletes (Borresen and Lambert, 2008; Lamberts et al., 2009; Daanen et al., 2012). For example, HRV kinetics during submaximal (D'Agosto et al., 2014) or maximal (Boullosa et al., 2012) exercise may be predictive of aerobic fitness and exercise performance. Similarly, HRV recovery following exercise occurs more rapidly in individuals with greater aerobic fitness (Stanley et al., 2013).

However, exercise can be performed in a multitude of different forms, including “aerobic” exercise (dynamic rhythmic exercise involving a large muscle mass, e.g., running and cycling), resistance exercise (e.g., weight/resistance training) as well as other forms (e.g., non-rhythmic/stochastic exercise, mixed-mode exercise, yoga, etc.), which may all elicit different effects on cardiac autonomic activity and HRV measures. Furthermore, these different types are each characterized by multiple sub-divisions that may be considered to constitute the exercise “dosage.” The focus of this review is on dynamic “aerobic” exercise as this form has received the most attention regarding HRV responses and is commonly used for exercise stress tests. The American College of Sports Medicine (ACSM) states that an acute bout of aerobic exercise can be modified by three primary factors constituting the exercise dose: intensity, duration, and modality (Pollock et al., 1998). If HRV responses to exercise and post-exercise recovery are to be interpreted with any diagnostic/prognostic value, it is important to establish how these factors of exercise prescription influence the response.

The controversies regarding the interpretation of HRV as reflecting cardiac sympathetic activity mean that HRV measures are not universally accepted to provide sympathetic insight (Eckberg, 1997; Billman, 2013b). Alternatively, systolic time intervals (STI), in particular the pre-ejection period (PEP), are demonstrated to reflect cardiac sympathetic influences on myocardial contractility (Harris et al., 1967; Ahmed et al., 1972; Cacioppo et al., 1994). Thus, monitoring the response of STI measures during exercise and recovery may provide insights into cardiac sympathetic activity (inotropic influences) to complement HRV measures of cardiac parasympathetic modulation (chronotropic influences). Accordingly, as for HRV, it is important to establish how the exercise dose affects the STI response to exercise and recovery.

Therefore, the purpose of this review is to: (a) summarize relevant literature relating to cardiac autonomic control during exercise and recovery; (b) present relevant background information on the measurement and interpretation of HRV; (c) examine and summarize the existing literature regarding how key exercise dose factors (intensity, duration, and modality) influence HRV responses to dynamic “aerobic” exercise, in particular during post-exercise recovery; (d) examine and summarize the existing literature regarding STI responses to exercise and recovery.

## Cardiac autonomic regulation during exercise and recovery

**Figure 1:**
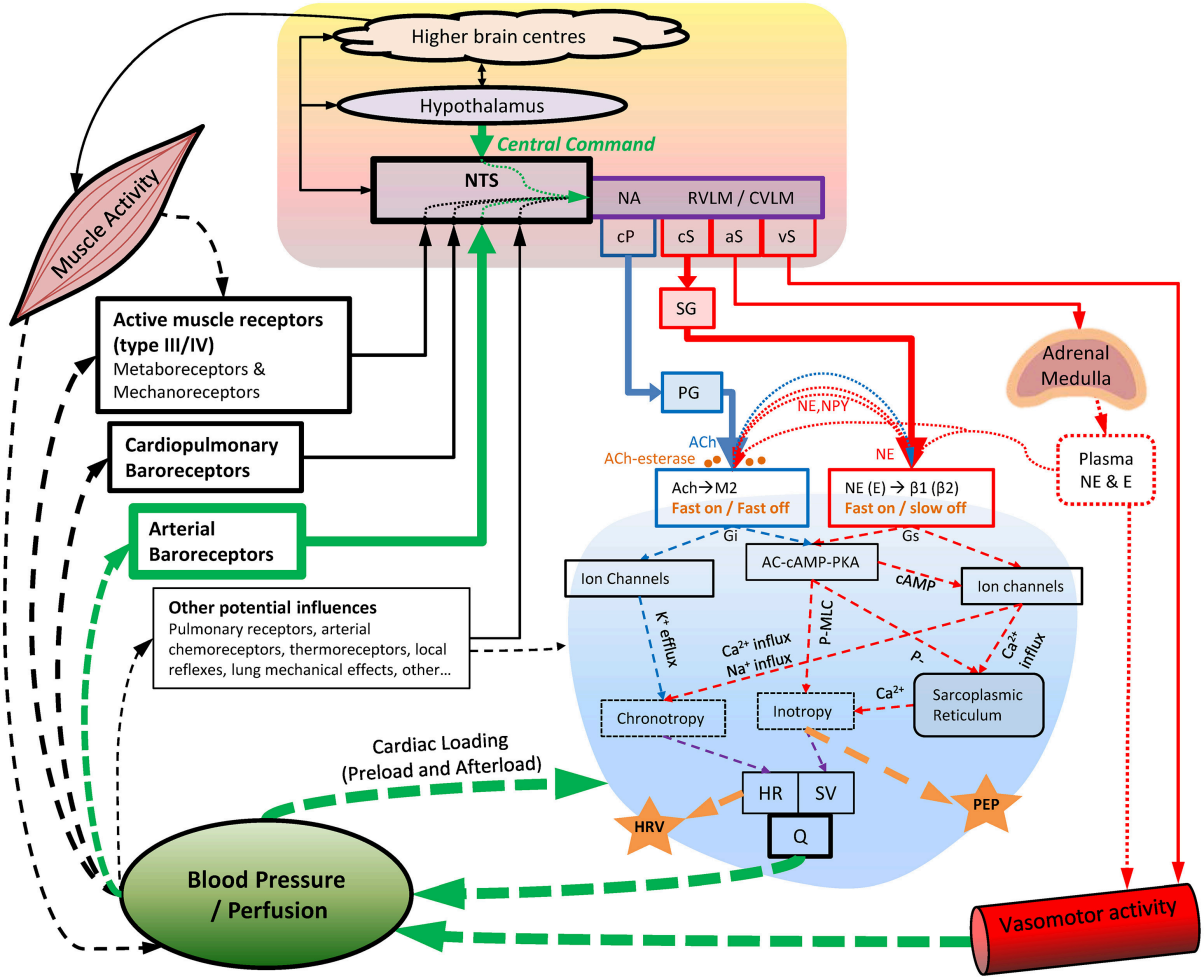
**Key aspects of cardiovascular autonomic regulation, particularly during exercise and recovery**. Blood pressure appears to be the primary regulated variable. Acetylcholine-esterase at the parasympathetic-cardiac junction facilitates rapid “On” and “Off” signaling (<1 s), whereas sympathetic “Off” effects are substantially slower (20+ s). Note the indirect nature of HRV and PEP as indices of cardiac parasympathetic and sympathetic activity, respectively, as well as the substantial “cross talk” (pre-and post-junctional) of cardiac sympathetic/parasympathetic effects. Also note the common pathways through which different dosages of exercise (intensity, duration, and modality) may influence cardiac autonomic regulation. AC-cAMP-PKA, adenylate-cyclase/cyclic-AMP/Protein-kinase-A cascade; ACh, acetylcholine; aS, sympathetic outflow to adrenal medulla; β1 (β2), Beta1 (Beta2) adrenergic receptors; Ca^2+^, calcium ions; cP, cardiac parasympathetic outflow; cS, cardiac sympathetic outflow; CVLM, caudal ventrolateral medulla; E, epinephrine; Gi, G-protein inhibitory subunit; Gs, G-protein stimulatory subunit; HR, heart rate; HRV, heart rate variability; K^+^, potassium ions; M2, M2 muscarinic receptor; MLC, myosin light chain; NA, nucleus ambiguus; Na^+^, sodium ions; NE, norepinephrine; NPY, neuropeptide Y; NTS, Nucleus Tractus Solitarii; P-, phosphorylation; PEP, pre-ejection period; PG, parasympathetic ganglia; Q, cardiac output; RVLM, rostro ventrolateral medulla; SG, sympathetic ganglia; SV, stroke volume; vS, vascular sympathetic outflow.

**Figure 2:**
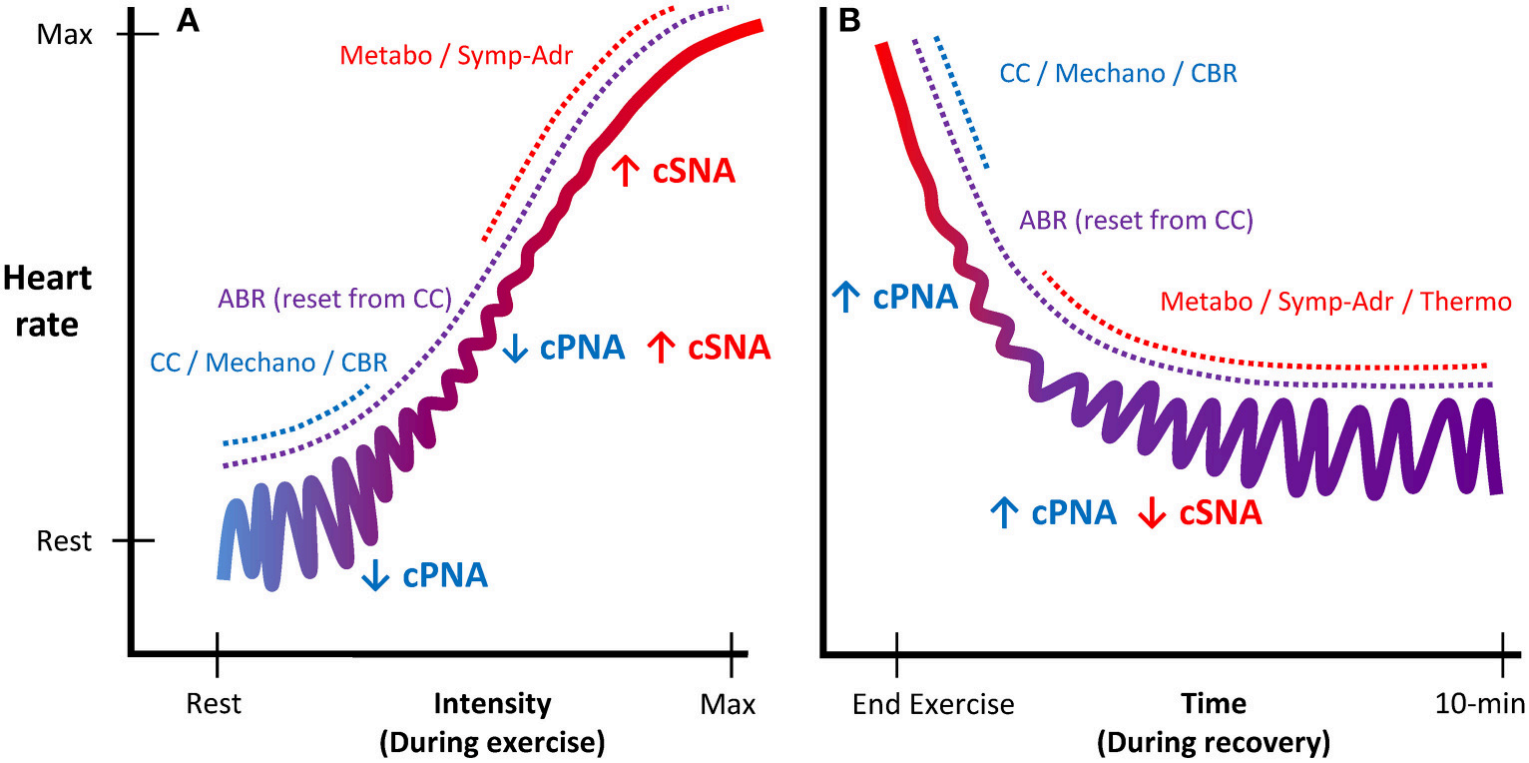
**Schematic illustration of autonomic regulation of HR during exercise and recovery**. Panel **(A)** displays HR regulation during exercise as a function of intensity. Panel **(B)** displays HR regulation during recovery as a function of time. As exercise intensity increases, cardiac control shifts from predominantly parasympathetic control (blue, acting as a “rapid modulator”) to predominantly sympathetic control (red, acting as a “tone-setter”). During recovery, the mechanisms eliciting cardio-acceleration during exercise are reversed, as HR regulation is gradually shifted back to predominantly parasympathetic control. cPNA, cardiac parasympathetic neural activity; cSNA, cardiac sympathetic neural activity; CC, central command; Mechano, mechanoreflex; CBR, central baroreflexes; ABR, arterial baroreflex; Metabo, metaboreflex; Symp-Adr, sympatho-adrenal; Thermo, thermoregulatory influences.

During exercise, substantial cardiovascular adjustments must occur to meet the competing demands of working muscles (metabolic demands) and skin blood flow (thermoregulatory demands), while maintaining blood pressure and adequate perfusion to other organs. Although some of the underlying mechanisms of *in-vivo* cardiac autonomic regulation during exercise remain contested, a prevailing model has emerged (Raven et al., 2006; Nobrega et al., 2014; White and Raven, 2014; Fadel, 2015; Fisher et al., 2015; Michelini et al., 2015) following on from the foundational work of Rowell et al. (Rowell and Oleary, 1990; Rowell, 1993; Rowell et al., 1996) and others (Robinson et al., 1966; O'leary, 1993; Potts and Mitchell, 1998). This model (Figure 1) proposes that upon initiation of exercise, descending “feed-forward” inputs from higher brain centers (“central command”) into the medullary cardiovascular center reset the arterial baroreflex to a higher operating point, triggering a rapid HR increase which is primarily mediated by reduced cardiac parasympathetic neural activity (cPNA), i.e., “parasympathetic withdrawal.” Rapid feedback from muscle mechanoreceptors contributes to initial parasympathetic withdrawal, while loading of the cardiopulmonary baroreceptors (due to an increase in venous return secondary to muscle pump action) likely also elicits cPNA withdrawal as well as an initial reduction in cardiac sympathetic neural activity (cSNA). Both cSNA and cPNA regulate HR throughout the entire exercise intensity continuum—cSNA working as a “tone-setter” and cPNA operating as a “rapid responder/modulator”—with the relative “balance” shifting from predominantly “parasympathetic control” at rest and low intensities to mainly “sympathetic control” at high intensities (White and Raven, 2014). As exercise intensity increases further, progressive baroreflex resetting as well as afferent feedback from muscle metaboreceptors trigger further cardiac parasympathetic withdrawal and sympathetic activation, the latter of which is increasingly augmented from moderate to maximal intensity by systemic sympatho-adrenal activation. These processes are summarized in Figure 2A.

Upon exercise cessation, the aforementioned processes mediating cardio-acceleration during exercise essentially occur in reverse. However, the details of which mechanisms mediate specific aspects of the post-exercise cardio-deceleration time-profile are to some extent less well-established, in part because of greater procedural variation (e.g., active vs. passive recovery and post-exercise posture). Nevertheless, the prevailing model (Imai et al., 1994; Kannankeril and Goldberger, 2002; Kannankeril et al., 2004; Pierpont and Voth, 2004; Coote, 2010; Pecanha et al., 2014) posits that abrupt removal of “central command” together with abolished feedback from muscle mechanoreceptors (for passive recovery) resets the arterial baroreflex to a lower level and causes an initial HR decrease, which is predominantly mediated by an increase in cPNA. Hence, this “fast phase” (i.e., initial minute) of HR recovery has often been attributed to “parasympathetic reactivation” (Perini et al., 1989; Imai et al., 1994; Cole et al., 1999; Coote, 2010; Pecanha et al., 2014), although some evidence has suggested sympathetic involvement as well (Nandi and Spodick, 1977; Kannankeril et al., 2004; Pichon et al., 2004). As recovery continues, a more gradual “slow phase” of cardio-deceleration is observed, likely mediated by both progressive parasympathetic reactivation and sympathetic withdrawal. These slower autonomic adjustments are believed to be elicited primarily by an intensity-dependent combination of gradual metabolite clearance (i.e., reduced metaboreflex input) and a reduction in circulating catecholamines, while thermoregulatory factors (direct thermoreceptor afferents and/or blood flow redistribution) may also be involved. These processes are summarized in Figure 2B.

## What is HRV and how is it quantified?

In 1996, the Task Force of The European Society of Cardiology and The North American Society of Pacing and Electrophysiology published a set of standards for the measurement and physiological interpretation of HRV (Camm et al., 1996). Briefly, as the name implies, HRV quantifies the variability of heart rate, although this is a misnomer as heart rate (beats per minute, b.min^−1^) is usually expressed as heart period (milliseconds per beat, ms) before variability is quantified. A key point is that HRV quantification involves several steps of calculations; each of these steps may be approached with different methodologies (with multiple sub-variations of these differing methodologies). As such, the field of HRV research is inherently heterogeneous from a methodological standpoint. Firstly, the length of time of data collection (epoch) varies greatly. Following data collection and removal/correction of signal artifacts and non-sinus beats, a detrending algorithm is often applied to minimize non-stationary aspects of the HR signal. Simple linear detrending is commonly employed (Camm et al., 1996), although higher-order or more complex algorithms have also been used (Tarvainen et al., 2002).

### Time-domain and frequency-domain analysis

Following data collection, correction, and detrending, the vast majority of HRV research employs time-domain and/or frequency-domain HRV measures. Regarding the time-domain (computationally the simplest and most consistent analysis method across studies), the two most common measures are the standard deviation of R-R intervals (SDRR), a measure of overall variability; and the root mean square of successive differences of R-R intervals (RMSSD), a measure of beat-to-beat variability. The latter is sometimes calculated slightly differently as the standard deviation of successive differences (SDsd).

Frequency-domain measures express HRV as a function of frequency, rather than time, since different spectral power components of HRV might relate to different elements of cardiac autonomic activity (Akselrod et al., 1985). There are different methods (and multiple sub-variations of methods) utilized to calculate HRV spectra, with the two most common employing a Fourier transform or autoregressive modeling. More recently, frequency-domain analysis has been applied to non-stationary signals using time-varying methods such as short-time Fourier transform (STFT) analysis (Cottin et al., 2006; Martinmaki et al., 2006; Kaikkonen et al., 2010, 2012). Regardless of the method used, the primary components are low frequency (LF, often 0.04–0.15 Hz) and high frequency (HF, often 0.15–0.40 Hz) spectra. Very low frequency spectra (VLF, <0.04 Hz) is seldom reported. Together these constitute total power (TP). These may be expressed as absolute power (ms^2^) or as power spectral density (ms^2^.Hz^−1^). Additionally, the ratio of LF to HF power (LF:HF) is also often reported, and HF and LF may also be normalized to TP (which may or may not include the VLF component) giving HF-nu and LF-nu (nu = normalized units). Variations of the upper and lower limits of each frequency band have also been employed (Radaelli et al., 1996; Avery et al., 2001; Pichon et al., 2004; Povea et al., 2005; Casties et al., 2006; Spadacini et al., 2006; Martinmaki et al., 2008).

Finally, HRV measures are often observed to be non-normally distributed. For this reason, a data transformation (typically natural-logarithm, Ln) is sometimes applied to yield an approximately normal distribution and permit parametric statistical analysis.

### Other methods of quantifying HRV

In addition to time and frequency domain HRV, other approaches of expressing the variability of HR are sometimes employed. Poincaré plots warrant mention because the measure “standard deviation 1” (SD1) is used to quantify short-term beat-to-beat HRV (similar to RMSSD). In fact, SD1 is a scaled derivative of SDsd (which is very similar to RMSSD), i.e., SD1 = SDsd ÷ √2 (Brennan et al., 2001). Indeed, in studies reporting SD1 and SDsd or RMSSD, manually dividing the reported SD1 results by √2 gives nearly identical results to those reported for SDsd (Tulppo et al., 1996) and RMSSD (Leicht et al., 2008). Other measures of HRV based on non-linear dynamics are less often utilized, such as power-law analysis, entropy, dimensionality and fractals (Oida et al., 1997; Huikuri et al., 2003, 2009; Perkiomaki et al., 2005; Baumert et al., 2009). Some reports have suggested that non-linear HRV measures may provide independent risk stratification and prognostic insight to complement time and frequency domain HRV measures (Makikallio et al., 1999; Huikuri et al., 2003; Stein et al., 2005), as well as offer certain advantages such as not requiring a stationary signal. In particular, fractal scaling exponents derived from detrended fluctuation analysis (DFA) might be of clinical value (Peng et al., 1995). However, Francis et al. (2002) suggested that fractal scaling exponents are fundamentally related to a weighted form of frequency-domain analysis. Regardless, the short term scaling exponent (α1) derived from DFA during incremental exercise may be modified by training (Karavirta et al., 2009). Moreover, it is interesting that the “inverted-U” bi-phasic response of DFAα1 during incremental exercise (Hautala et al., 2003) appears to be generally consistent with the bi-phasic nature of parasympathetic reflex control of HR as a function of exercise intensity (White and Raven, 2014). Notwithstanding the potential value of non-linear HRV measures (which warrant further investigation), these techniques are beyond the scope of this review.

### Physiological interpretation of HRV

Interpretation of HRV as reflecting certain aspects of cardiac autonomic activity is complicated by the fact that rather than being a direct measure of autonomic nerve activity, HRV quantifies the modulation of the end-organ response, i.e., HR. HR is under the dual control of direct sympathetic and parasympathetic innervation at the sino-atrial (SA) node, but is also influenced by sympatho-adrenal activation and non-autonomic effects such as mechanical/hemodynamic influences and local reflexes (Bernardi et al., 1990; Rowell et al., 1996). Furthermore, complex non-linear pre-junctional and post-junctional parasympathetic-sympathetic interactions have been reported (Levy, 1971; Kawada et al., 1996; Sunagawa et al., 1998; Uijtdehaage and Thayer, 2000; Miyamoto et al., 2003, 2004; Paton et al., 2005), whereby activity of one branch can in some instances augment and in other instances attenuate the activity and/or effect of the other branch. It is therefore critical to appreciate that HRV is an indirect indicator of cardiac autonomic modulation.

#### Interpretation of HRV measures associated with respiratory sinus arrhythmia

Despite the inherent limitations of HRV as an indirect measure of modulation of cardiac effect, oscillations in cPNA (secondary to respiration) are generally considered to be the primary contributor to HRV measures expressing beat-to-beat/rapid HR variations (Pomeranz et al., 1985; Randall et al., 1991; Malik and Camm, 1993; Camm et al., 1996; Goldberger et al., 2006). Parasympathetic blockade reduces these “cPNA-HRV measures” (e.g., RMSSD, SD1 and HF) in a dose-dependent manner (Tulppo et al., 1996; Medigue et al., 2001; Hautala et al., 2003). Furthermore, during exercise the response of these measures is generally consistent with current understandings of cardiac autonomic regulation, i.e., an increase in cSNA and a decrease in cPNA (Robinson et al., 1966; Rowell and Oleary, 1990; White and Raven, 2014). Under normal conditions these measures are gradually reduced during progressive exercise (Yamamoto et al., 1991; Tulppo et al., 1996, 1998; Hautala et al., 2003; Casties et al., 2006; Martinmaki et al., 2008; Karapetian et al., 2012), whereas this response is greatly reduced or abolished under parasympathetic blockade (Tulppo et al., 1996; Warren et al., 1997; Hautala et al., 2003).

However, cPNA-HRV measures are not exact quantitative measures of cardiac parasympathetic activity. Although greater parasympathetic activity is generally associated with greater modulation of parasympathetic effect, very high parasympathetic activity may lead to acetylcholine saturation at the SA node, thus decreasing HRV (Eckberg, 2003; Dewland et al., 2007). Also, while sympathetic activity tends to have lesser/minimal effects on these cPNA-HRV measures (Warren et al., 1997; Polanczyk et al., 1998; Tulppo et al., 1999; Ng et al., 2009), some influence must be acknowledged since sympathetic blockade may augment these measures (Taylor et al., 2001; Ng et al., 2009), possibly via a permissive effect due to decreased sympathetic inhibition of parasympathetic activity/effect. This highlights the importance of considering parasympathetic-sympathetic interactions (Figure 1) whenever interpreting measures of cardiac autonomic activity. During exercise, cPNA-HRV measures usually reach an intensity-dependent minimum at a moderate intensity, e.g., 50–60% maximal oxygen uptake (VO_2_max) (Tulppo et al., 1996; Yamamoto et al., 2001; Cottin et al., 2006), whereas cardiac parasympathetic activity may demonstrate a progressive decrease up to maximal exercise intensity (White and Raven, 2014). Again, it might be that despite small parasympathetic effects on HR even at maximal intensity (Kannankeril et al., 2004), beat-to-beat modulation of these effects above moderate intensity may be inhibited by strong sympathetic (cardiac nerve and sympatho-adrenal) activation.

#### Interpretation of other HRV measures

Measures of overall variability (e.g., SDRR and TP) are considered to be influenced by both parasympathetic as well as sympathetic cardiac activity, although these measures tend to be associated with cPNA-HRV measures (Tulppo et al., 1996; Warren et al., 1997; Hautala et al., 2003; Martinmaki et al., 2008), suggesting that parasympathetic activity is the primary influence. Although HF-nu has sometimes been employed as a cPNA-HRV index (Saito and Nakamura, 1995; Avery et al., 2001; Casonatto et al., 2011; Teixeira et al., 2011), the response of this measure during exercise is not consistent with a decrease in cardiac parasympathetic activity (Hautala et al., 2003; Casties et al., 2006; Leicht et al., 2008; Martinmaki and Rusko, 2008).

Some studies have suggested that LF, LF-nu, and LF:HF may reflect sympathetic activity or “sympatho-vagal balance” (Pagani et al., 1986; Malliani et al., 1991; Yamamoto et al., 1991; Saito and Nakamura, 1995; Avery et al., 2001; Teixeira et al., 2011), although this is controversial (Cacioppo et al., 1994; Eckberg, 1997; Warren et al., 1997; Billman, 2006, 2013b). In particular, parasympathetic blockade greatly attenuates LF (Cacioppo et al., 1994; Warren et al., 1997; Hautala et al., 2003), suggesting strong parasympathetic influence. Furthermore, under conditions that would be expected to increase cardiac sympathetic activity, such as exercise (Robinson et al., 1966; White and Raven, 2014) and myocardial ischemia (Houle and Billman, 1999), a decrease in LF is often observed (Arai et al., 1989; Yamamoto et al., 1991; Casadei et al., 1995; Houle and Billman, 1999; Hautala et al., 2003; Casties et al., 2006; Martinmaki and Rusko, 2008; Martinmaki et al., 2008). Thus, although sympathetic activity does contribute to LF, parasympathetic activity appears to be a stronger influence (Pomeranz et al., 1985; Randall et al., 1991; Cacioppo et al., 1994). There is also a strong parasympathetic influence on ratio measures (LF-nu and particularly LF:HF; Cacioppo et al., 1994), likely because HF (predominantly reflecting respiratory sinus arrhythmia) is directly used in the calculation of these measures. Furthermore, inconsistent responses to stressors which would increase sympathetic activity have been observed (Radaelli et al., 1996; Hautala et al., 2003; Casties et al., 2006; Martinmaki and Rusko, 2008), although generally a decrease has been reported during higher intensity exercise and thus they are not consistent with sympathetic activity/dominance. Additionally, the concept of “sympatho-vagal balance” has been challenged at a conceptual level (Eckberg, 1997; Billman, 2013b; White and Raven, 2014), in particular the underlying assumptions that sympathetic activity is a key contributor to LF and that sympathetic and parasympathetic activity/effects operate in a reciprocal manner with linear interactions. Despite these controversies, LF:HF is often employed measure of “sympatho-vagal balance,” where supposedly an increase indicates “sympathetic dominance” and a decrease indicates “parasympathetic dominance.”

### Summary—HRV background and physiological interpretation

For clinicians and exercise/sport scientists, the primary interest in HRV relates to (a) its prognostic potential value in cardiac disease and sudden cardiac death (Camm et al., 1996; Tsuji et al., 1996; Kikuya et al., 2000), and (b) the general acceptance of beat-to-beat measures (cPNA-HRV) as indicators of cardiac parasympathetic modulation. Indeed, notwithstanding some interpretative controversies, these measures are regularly employed in this manner. The underlying mechanisms and interpretation of other HRV measures (such as LF, LF-nu, and LF:HF) are less established, likely resulting from complex sympathetic-parasympathetic interactions. While this has not prevented the use of these measures as indices of sympathetic activity or “sympatho-vagal balance,” the majority of evidence does not support this approach. In light of interpretative controversies, cPNA-HRV measures perhaps provide qualitative or ordinal (rather than parametric quantitative) insight into cardiac parasympathetic activity (Billman, 2006).

## HRV during exercise

Several studies have investigated HRV during exercise (Bernardi et al., 1990; Perini et al., 1990, 2000; Yamamoto et al., 1991, 1992; Dixon et al., 1992; Radaelli et al., 1996; Tulppo et al., 1996, 1998, 1999; Shibata et al., 2002; Casties et al., 2006; Cottin et al., 2006, 2007; Karapetian et al., 2008; Kaikkonen et al., 2010). However, in addition to widely varying HRV analysis methodologies amongst the HRV literature, studies employing exercise with HRV measurements also vary markedly in terms of the participants and exercise protocol. Studies have used a range of exercise modalities, with cycling the most common mode employed (Yamamoto et al., 1991; Radaelli et al., 1996; Tulppo et al., 1996, 1998; Hautala et al., 2003; Casties et al., 2006; Cottin et al., 2006; Karapetian et al., 2008; Martinmaki and Rusko, 2008; Martinmaki et al., 2008), although walking/running has also been utilized (Hautala et al., 2003; Cottin et al., 2007; Botek et al., 2010; Kaikkonen et al., 2010). Other modes less commonly used are arm-cranking (Tulppo et al., 1999; Leicht et al., 2008), rowing (Cheng et al., 2005) and swimming (Di Michele et al., 2012). Regarding the effect of exercise dose factors (intensity, duration, and modality), intensity has received the most investigative attention, while fewer studies have investigated the effects of duration and modality.

### Effect of exercise intensity

Several studies have investigated the effect of exercise intensity on HRV during exercise. In addition to different modalities being utilized between these studies, the duration for which participants exercised at each intensity varies greatly, such as 2 min (Tulppo et al., 1996; Martinmaki et al., 2008), 3 min (Tulppo et al., 1998, 1999; Hautala et al., 2003; Karapetian et al., 2012), 5 min (Radaelli et al., 1996), 8 min (Casties et al., 2006; Michael et al., 2016), 10 min (Martinmaki and Rusko, 2008) and 15 or more min (Yamamoto et al., 1991; Saito and Nakamura, 1995; Leicht et al., 2008; Boettger et al., 2010). Nevertheless, an analysis of the literature allows us to identify some general responses for some HRV measures as a function of exercise intensity.

#### Time-domain measures

Exercise elicits a reduction in HRV when expressed in the time domain, regardless of whether the HRV metric is based on overall HRV calculated from R-R intervals (e.g., SDRR), or beat-to-beat HRV metrics based on the difference in R-R intervals (e.g., RMSSD). Several studies have reported that higher exercise intensity is associated with a lower SDRR (Yamamoto et al., 1991; Saito and Nakamura, 1995; Radaelli et al., 1996; Tulppo et al., 1996; Hautala et al., 2003; Casties et al., 2006; Spadacini et al., 2006; Leicht et al., 2008; Fisher et al., 2009; Karapetian et al., 2012). Compared to resting values which vary greatly (typically 40–100 ms), there is a somewhat consistent intensity dose-response, namely a curvilinear decay to 3–10 ms for exercise >160 b.min^−1^. The results of some studies indicate that SDRR reaches a minimum at moderate-high intensity, and does not change substantially thereafter, whereas other studies suggest that small decreases in SDRR continue as intensity is increased toward maximal. Regardless, it is clear that SDRR is drastically reduced as a function of exercise intensity. When natural-log transformed, SDRR (Ln-SDRR) demonstrates a somewhat linear decrease as a function of exercise intensity.

**Figure 3:**
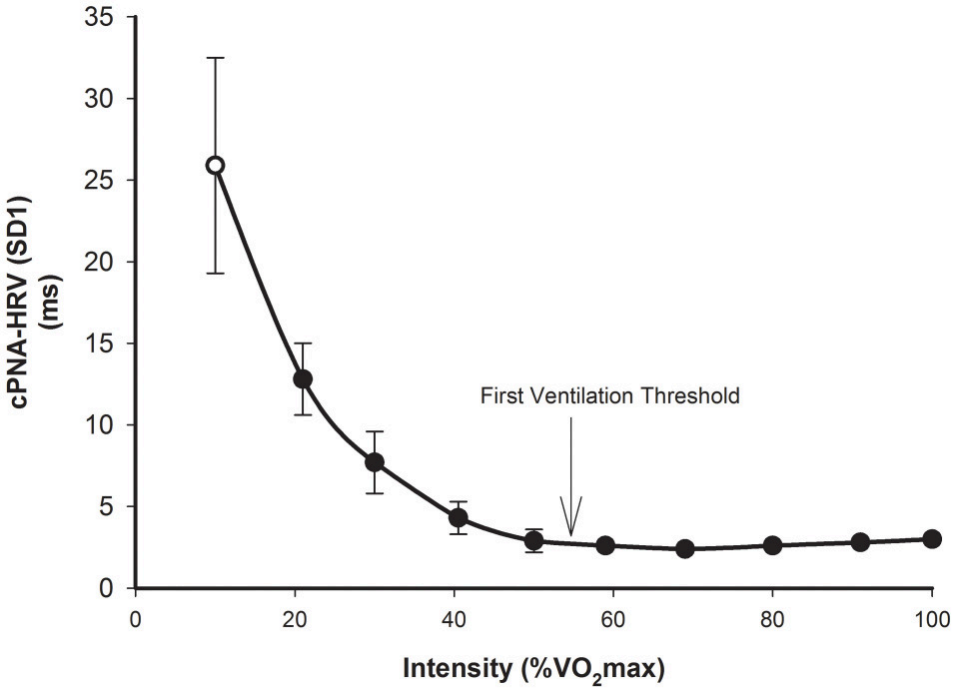
**cPNA-HRV (SD1 from Poincaré plot, ms) during rest (hollow) and incremental exercise (filled)**. Data are mean ± SD. Redrawn from Tulppo et al. (1996).

The basic response of cPNA-HRV (e.g., RMSSD) is similar to that of SDRR, i.e., a higher exercise intensity is associated with a lower RMSSD, and that this follows a relatively consistent curvilinear decay profile as a function of exercise intensity (Tulppo et al., 1996; Povea et al., 2005; Leicht et al., 2008; Fisher et al., 2009; Boettger et al., 2010; Lunt et al., 2011; Karapetian et al., 2012). The intensity-dependent reduction in RMSSD occurs more rapidly than compared with SDRR, such that a minimum value (<5 ms) is reached at a moderate intensity, i.e., ~120–140 b.min^−1^, or 50–60% VO_2_max. Thereafter, RMSSD does not change substantially, although slight increases have sometimes been observed at higher exercise intensities (e.g., >180 b.min^−1^). As discussed in the previous section, SD1 (from Poincaré plots) is essentially a scaled version of RMSSD, thus it is not surprising that the intensity dose-response for SD1 (Figure 3) is the same as that of RMSSD (Tulppo et al., 1996; Leicht et al., 2008; Garcia-Tabar et al., 2013). When these measures are expressed relative to the underlying RRI, which has been suggested to minimize the purely mathematical influence of HR on HRV (Sacha, 2013, 2014; Pradhapan et al., 2014), the same intensity dose-response decay profile is maintained (Tulppo et al., 1998, 1999). When natural-log transformed, RMSSD (Ln-RMSSD) demonstrates an approximately linear decrease until moderate-high intensities (140–160 b.min^−1^), followed by no change or a small increase at higher intensities.

Regarding practical application, the point at which beat-to-beat cPNA-HRV measures such as RMSSD and SD1 reach a minimum has been termed a “HRV threshold” (HRVT). The HRVT appears to be a proxy for determining the intensity corresponding to the first ventilation threshold (VT1) or first lactate threshold (Karapetian et al., 2008, 2012; Sales et al., 2011; Garcia-Tabar et al., 2013), thus making it a potentially useful tool in exercise testing/monitoring and training prescription.

#### Frequency domain measures

Similar to when expressed in the time domain, the frequency domain measures of HRV also demonstrate a pronounced reduction in response to exercise. While the conventional HF band of 0.15–0.40 Hz has often been employed during exercise (Tulppo et al., 1996, 1998; Hautala et al., 2003; Leicht et al., 2008; Fisher et al., 2009; Boettger et al., 2010), this band may not be suitable during exercise where higher respiratory frequencies are observed, and therefore different lower limits such as 0.18 Hz have previously been employed (Radaelli et al., 1996; Spadacini et al., 2006), as have different upper limits including 0.35 Hz (Spadacini et al., 2006), 0.50 Hz (Dixon et al., 1992; Avery et al., 2001; Povea et al., 2005), 0.80 Hz (Lunt et al., 2011), 1.00 Hz (Pichon et al., 2004; Casties et al., 2006; Martinmaki and Rusko, 2008), 1.20 Hz (Martinmaki et al., 2008), 1.50 Hz (Perini et al., 2006; Michael et al., 2016), and 2.00 Hz (Cottin et al., 2006). Furthermore, most studies report absolute power (ms^2^) Yamamoto et al., 1991; Saito and Nakamura, 1995; Tulppo et al., 1996; Hautala et al., 2003; Pichon et al., 2004; Spadacini et al., 2006; Leicht et al., 2008; Martinmaki and Rusko, 2008; Martinmaki et al., 2008; Fisher et al., 2009, although some report power spectral density (ms^2^.Hz^−1^) (Avery et al., 2001; Povea et al., 2005; Casties et al., 2006). Several studies report raw measures (Yamamoto et al., 1991; Saito and Nakamura, 1995; Tulppo et al., 1996; Avery et al., 2001; Hautala et al., 2003; Pichon et al., 2004; Casties et al., 2006; Leicht et al., 2008; Fisher et al., 2009), while others report natural log transformed measures (Radaelli et al., 1996; Spadacini et al., 2006; Martinmaki and Rusko, 2008; Martinmaki et al., 2008; Kaikkonen et al., 2010; Lunt et al., 2011). Another discrepancy occurs when considering studies investigating normalized power, as the total power to which measures are normalized may (Saito and Nakamura, 1995; Warren et al., 1997; Povea et al., 2005; Boettger et al., 2010) or may not (Hautala et al., 2003; Pichon et al., 2004; Casties et al., 2006; Leicht et al., 2008; Martinmaki and Rusko, 2008) include the VLF component.

Despite the heterogeneity regarding the signal analysis methodology of frequency domain HRV, some common trends are observed. As for time-domain measures, LF, HF, and TP all demonstrate a substantial decay-type decrease with increasing exercise intensity, up to a particular intensity after which minimal spectral power remains and no further decrease is observed. The intensity above which no further decline is observed varies greatly, but is usually in the range of 120–180 b.min^−1^ (Radaelli et al., 1996; Tulppo et al., 1996; Avery et al., 2001; Hautala et al., 2003; Povea et al., 2005; Casties et al., 2006; Spadacini et al., 2006; Martinmaki et al., 2008; Fisher et al., 2009). As for beat-to-beat time domain measures, some studies have demonstrated that analysis of the HF decay with incremental exercise may serve as a surrogate method for determining the intensity associated with first ventilation threshold (Cottin et al., 2006, 2007). Furthermore, HF power multiplied by the HF frequency may provide an approximation for the intensity associated with the second ventilation threshold (Cottin et al., 2006, 2007; Buchheit et al., 2007b).

Considering the relationship between normalized measures of LF and HF (usually relative to LF+HF or VLF+LF+HF), it is not surprising that these measures behave in an opposite manner to each other during exercise. Typically, LF-nu increases during low-moderate intensity exercise and decreases during higher intensity exercise, while HF-nu demonstrates the opposite response (Bernardi et al., 1990; Perini et al., 1990, 1993, 1998; Hautala et al., 2003; Pichon et al., 2004; Povea et al., 2005; Martinmaki and Rusko, 2008). However, conflicting responses have also been reported (Perini et al., 1993; Avery et al., 2001; Casties et al., 2006; Boettger et al., 2010). LF:HF demonstrates inconsistent responses to exercise. Some studies reported an increase until low-moderate intensity (110–130 b.min^−1^), followed by a decrease during higher intensities (Radaelli et al., 1996; Tulppo et al., 1996; Hautala et al., 2003). However, other studies reported a progressive decrease from rest with increasing exercise intensity (Casties et al., 2006), a progressive increase from rest (Saito and Nakamura, 1995; Avery et al., 2001), or minimal change at low-moderate intensity followed by a sharp increase at moderate intensity (Yamamoto et al., 1991). Some of these divergent findings may be attributed to varying methodology (especially HRV analysis techniques). However, it is interesting that these normalized and ratio measures rarely (if ever) behave in a manner that is consistent with the established exercise response of systems they purportedly reflect—namely cardiac parasympathetic activity (HF-nu) and sympathetic activity or sympatho-vagal balance (LF-nu and LF:HF).

### Effect of exercise duration

Compared to the effect of exercise intensity, few studies have investigated the effect of exercise duration HRV responses during exercise. This may be in part due to the pronounced effect of intensity on HRV, which has two important implications for investigating other influences on HRV during exercise: (1) Due to the HRV-intensity relationship (whereby most HRV measures reach a near-zero minimum at/above moderate intensity), any potential influence of duration is minimized; and (2) During prolonged exercise HR may change despite no change in external load, i.e., cardiovascular drift (Montain and Coyle, 1992), meaning that “internal intensity” is important to consider. Thermoregulatory factors (in particular fluid losses from sweating) play a key role in cardiovascular drift as exercise is prolonged (Coyle and Gonzalez-Alonso, 2001), with significant cardiovascular autonomic implications due to changes in blood pressure and blood flow redistribution (Figure 1).

Kaikkonen et al. (2007) investigated the effect of exercise duration on frequency domain HRV measures during different exercise intensities in sedentary women, who performed low intensity (~45% VO_2_max) and high intensity (~77% VO_2_max) exercise for two different durations (approximately 38 vs. 76 min for low intensity and 30 vs. 60 min for high intensity). Despite a doubling of exercise duration, no significant difference in HRV during exercise was observed for either intensity. Similar findings were reported by the same authors in a later study (Kaikkonen et al., 2010) investigating the effect of a 300–400% increase in exercise duration, in which recreational-level athletes ran at ~66% VO_2_max for ~20 or ~90 min.

Pichon et al. (2004) investigated frequency domain HRV measures during different short-duration exercise bouts, i.e., 3, 6, and 9 min of exercise at 60 and 70% of power reached at VO_2_max (PVO_2_max), and 3 and 6 min at 80% PVO_2_max. HF was higher during 3 min compared to 6 and 9 min. LF also decreased with increased exercise duration. No significant effects were observed for normalized measures or LF:HF. However, as all three intensities were of at least moderate-high intensity, the heart rates differed substantially between exercise durations. The lowest intensity (60% VO_2_max) elicited a mean HR of 143, 161, and 167 b.min^−1^ at 3, 6, and 9 min, respectively. These differences were enhanced with higher intensities, likely in part because of exercise onset HR kinetics and the time required to reach steady state. Thus, despite these bouts being matched for relative intensity (in terms of %PVO_2_max), the elevated HR associated with higher intensity exercise of such short durations means it is difficult to separate the independent effect of exercise duration from that of exercise intensity in this study. Similarly, Moreno et al. (2013) investigated time and frequency domain HRV throughout 90 min of moderate intensity exercise (60% VO_2_peak). Despite HRV measures being greatly reduced by the time of the initial exercise recording (25 min), the authors found that HR increased throughout 90 min of exercise (from ~140 to ~150 b.min^−1^), while SDRR, RMSSD, LF and HF were further decreased by 90 min.

### Effect of exercise modality

Relatively few studies have compared the HRV responses during different modalities of dynamic exercise. In addition to the methodological challenge involving the strong HRV-intensity relationship, it is difficult to standardize exercise “intensity” when comparing different modalities. Some of these issues relate to what basic metric to employ (e.g., power output, HR, or VO_2_). For example, arm crank exercise will elicit a higher HR when compared with leg cycling for the same rate of energy expenditure (same absolute VO_2_; Tulppo et al., 1999) or work rate (Leicht et al., 2008). There is also the issue of whether to match relative or absolute intensity (and whether this is mode-specific). For instance, peak HR will likely be 10–20 b.min^−1^ lower during arm cranking compared with leg cycling (Tulppo et al., 1999; Ranadive et al., 2011). The physiological response (e.g., HR or VO_2_) elicited for any intensity of arm crank exercise is likely not equal to response elicited during leg cycling exercise, despite the workload representing the same percentage of mode-specific maximum HR or maximum VO_2_. Because of these inherent methodological challenges, the effect of modality on HRV during exercise has been investigated with varying approaches.

#### Different modes of dynamic exercise

Tulppo et al. (1999) investigated HRV during incremental arm crank compared to incremental cycling exercise using SD1 and SD1n (SD1 divided by RRI) as parasympathetic measures. As expected for both modes of exercise, these measures decreased as intensity increased until a moderate power output (~50% VO_2_max) was reached. Below this power output, HR was higher and HRV was lower during arm exercise compared with leg exercise. The authors concluded that incremental arm exercise results in a more rapid vagal withdrawal compared with incremental leg exercise. Interestingly, manual re-plotting of the data reveals that when HRV is plotted against HR (rather than VO_2_), there appears to be no difference between the modalities. These findings were in contrast to those of Leicht et al. (2008), who investigated HRV responses during lower body exercise (cycling), upper body exercise (arm crank), and whole body exercise (running) matched for HR. Absolute VO_2_ was similar during cycling and running but lower during arm crank, whereas perceived exertion was higher during arm crank. Despite HR being matched, time and frequency domain measures were similar during cycling and running, but were higher during arm crank, thus demonstrating that exercise mode can affect HRV, independent of the underlying HR. Whilst methodological differences may explain part of these apparently discrepant findings, it is nonetheless clear that exercise intensity is a stronger determinant of HRV response to exercise than modality.

A small number of studies have investigated HRV during different conditions/modalities that are likely to elicit different circulatory/orthostatic effects. Di Rienzo et al. (2008) compared normal cycling to 0-g cycling (in space) at 75W, though no significant differences in HR or HRV (time and frequency domain) were observed. Somewhat comparable are the findings of Perini et al. (1993), who compared upright vs. supine cycling at different absolute workloads (50, 100, 150 W). RRI and TP both decreased with increasing intensity in a similar manner for both postures (although the normalized powers for VLF, LF, and HF varied as a function of intensity and posture). Takahashi et al. (2000) investigated HRV responses in older adults during incremental treadmill exercise on land and whilst immersed in water, with HF amplitude (rather than power) utilized as a measure of cardiac vagal outflow. HR differed during incremental exercise between the two conditions (lower in water at low intensities, then higher in water at high intensities, compared to land) although no significant difference was found in HF amplitude at any intensity. Similar findings were reported by Perini et al. (1998), who investigated the effect of water immersion on HRV responses to incremental cycling exercise and found that HRV responses were similar between the two conditions throughout incremental exercise. Thus, if these orthostatic conditions had any influence on HRV during exercise, the effects were minor when compared with the strong influence of exercise intensity.

#### Other “mode” comparisons

Although the focus of this review is on dynamic “aerobic” exercise, there have been some studies comparing the HRV responses amongst other “modes” of exercise that warrant mention, particularly considering the scarcity of research involving aerobic exercise modes and HRV. Cottin et al. (2004) investigated HRV measures during two different high intensity exercises, namely dynamic (cycling) vs. irregular (Judo Randori). HR was similar during both exercises (above 180 b.min^−1^), although frequency domain and Poincaré HRV measures were higher during Judo. Due to the high heart rates, the physiological interpretation of these findings is difficult, as non-neural mechanisms such as respiration are believed to substantially influence HRV at such high intensities (Casadei et al., 1995, 1996; Cottin et al., 2006, 2007). Furthermore, the metabolic drive to HR vs. the hormonal stress anticipatory response of combat activity (Salvador et al., 2003) represent different aspects of “modality” that are not easily accounted for or compared.

Gonzalez-Camarena et al. (2000) compared static exercise (isometric knee extension at 30% MCV) to dynamic exercise (cycling at 30 and 60% VO_2_max). HRV (time and frequency domain measures) was reduced during dynamic exercise whereas an increase was observed during static exercise, although HR was not matched between exercises. Similar finding were reported by Weippert et al. (2013, 2015), who compared HRV during static exercise (supine isometric leg press) vs. dynamic exercise (supine cycling) at similar low-intensity heart rate levels (~88 b.min^−1^). HRV measures of overall variability (SDRR) as well as beat-to-beat variability purportedly reflecting cardiac parasympathetic activity (RMSSD, HF, SD1) were higher during static exercise compared to dynamic exercise (as was subject effort, blood pressure, and rate-pressure product).

### Reliability of HRV during exercise

A small number of studies have investigated the test-retest reliability of HRV measures during exercise. Apart from aforementioned disparities amongst the literature regarding exercise protocols and HRV analysis methodology, there are also differences in the assessment of reliability. The intraclass correlation coefficient (ICC) reflects the ability of a test to differentiate between individuals and is therefore considered a measure of relative reliability, although there are multiple methods of calculating ICC (Weir, 2005). Alternatively, measures such as the coefficient of variation (CV), typical error of measurement (TEM, which may be expressed as a CV), and Bland-Altman limits of agreement (LoA) may be considered measures of absolute reliability as they reflect the trial-to-trial noise. Carrasco et al. (2003) demonstrated that the ICC for a range of time and frequency domain measures (Ln-transformed) during cycling exercise at 80 W ranged between 0.70 and 0.91, which was comparable to resting measures. Similar ICC-values (~0.9) were reported for Ln-transformed time domain measures during slow walking at 4 km/hr in another study (Boullosa et al., 2014). These authors also reported that that the TEM(CV) was 16–22%. Utilizing a Bland-Altman approach, Tulppo et al. (1998) demonstrated that the LoA were lower during cycling exercise for time and frequency domain measures compared to rest, although this may be partly due to the substantially reduced values during exercise compared with rest.

### Summary—HRV during exercise

During exercise, HRV measures demonstrate a curvilinear decay as a function of exercise intensity, that is closely related to exercising HR. HRV measures associated with cardiac parasympathetic activity (e.g., RMSSD and HF) usually reach a near-zero minimum at moderate intensity (possibly being associated with the first ventilation/lactate threshold). These measures are sometimes observed to increase slightly as exercise intensity increases toward maximum, although this is likely mediated by non-neural mechanisms such as direct mechanical effects of respiration on the SA node. The data also leads to further questioning of the use of frequency domain ratio and normalized measures as indicators of sympathetic activity or “sympatho-vagal balance.” In addition to demonstrating inconsistent responses to exercise, the response of these measures is rarely consistent with our current understanding of autonomic control during exercise, namely progressive parasympathetic withdrawal and sympathetic activation (White and Raven, 2014).

Regarding exercise duration, the limited body of literature suggests that prolonged exercise duration can influence (attenuate) HRV during exercise, although this has only been observed in studies where there is a concomitant increase in HR (i.e., cardiovascular drift) and also when HRV has not already reached the intensity-dependent minimum. Indeed, this elevated HR (representing elevated internal intensity, regardless of the external load) may be the cause for any change in HRV, rather than any direct effect of exercise duration. Conversely, prolonged exercise duration may be associated with progressive parasympathetic withdrawal (indicated by lower HRV), and this in turn might contribute to cardiovascular drift (Kukielka et al., 2006). Regardless of the direction of any cause-effect relationship, it is difficult to infer what affects HRV under conditions of different HR-values, as HR likely has a purely mathematical effect on HRV (Billman, 2013a; Sacha, 2013, 2014), whereby a greater HR can reduced HRV despite no change in the actual variability of autonomic outflow.

While there is limited data available, the evidence suggests that exercise modality can modify HRV, although not all research supports this. Interpretation of results is difficult as any influence of exercise modality is confounded by the issue of matching for exercise “intensity.” Interestingly, studies investigating different dynamic modalities that would be expected to elicit different orthostatic/circulatory conditions (e.g., posture, gravity, or water immersion) have typically found no substantial effect on HRV during exercise. Instead, the muscle group employed and the mode of contraction (e.g., static vs. dynamic) seem to be stronger modality-related factors influencing HRV response during exercise. Nevertheless, exercise intensity is the strongest determinant of HRV during exercise.

## HRV during post-exercise recovery

Most HRV measures are substantially reduced during exercise (see previous section). HRV has also been employed as a tool to investigate post-exercise autonomic (predominantly parasympathetic) activity (Goldberger et al., 2006; Buchheit et al., 2007a, 2009a; Al Haddad et al., 2010; Stanley et al., 2013; Ahmadian et al., 2015). Upon exercise cessation, HR and HRV demonstrate a time-dependent recovery and eventual return to pre-exercise levels (Stanley et al., 2013). Recovery conditions such as posture have also been shown to affect HRV recovery (Barak et al., 2010), with a more upright posture slowing recovery (Buchheit et al., 2009a). Rapid (though incomplete) recovery is commonly observed in the initial minutes following exercise (Buchheit et al., 2007a, 2009b; Kaikkonen et al., 2007, 2010, 2012; Martinmaki and Rusko, 2008; Al Haddad et al., 2011; Stanley et al., 2013). While complete recovery may take up to 48 h following some bouts of exercise and may sometimes involve an “overshoot” above pre-exercise levels prior to 48 h (Furlan et al., 1993; Hautala et al., 2001; Stanley et al., 2013), the focus of this review is on the immediate post-exercise recovery period (e.g., 0–10 min). As is the case during exercise, the majority of studies investigating the effect of exercise on HRV recovery have focused on different exercise intensities, while fewer studies have investigated the effects of exercise duration or modality.

### Effect of exercise intensity

**Figure 4:**
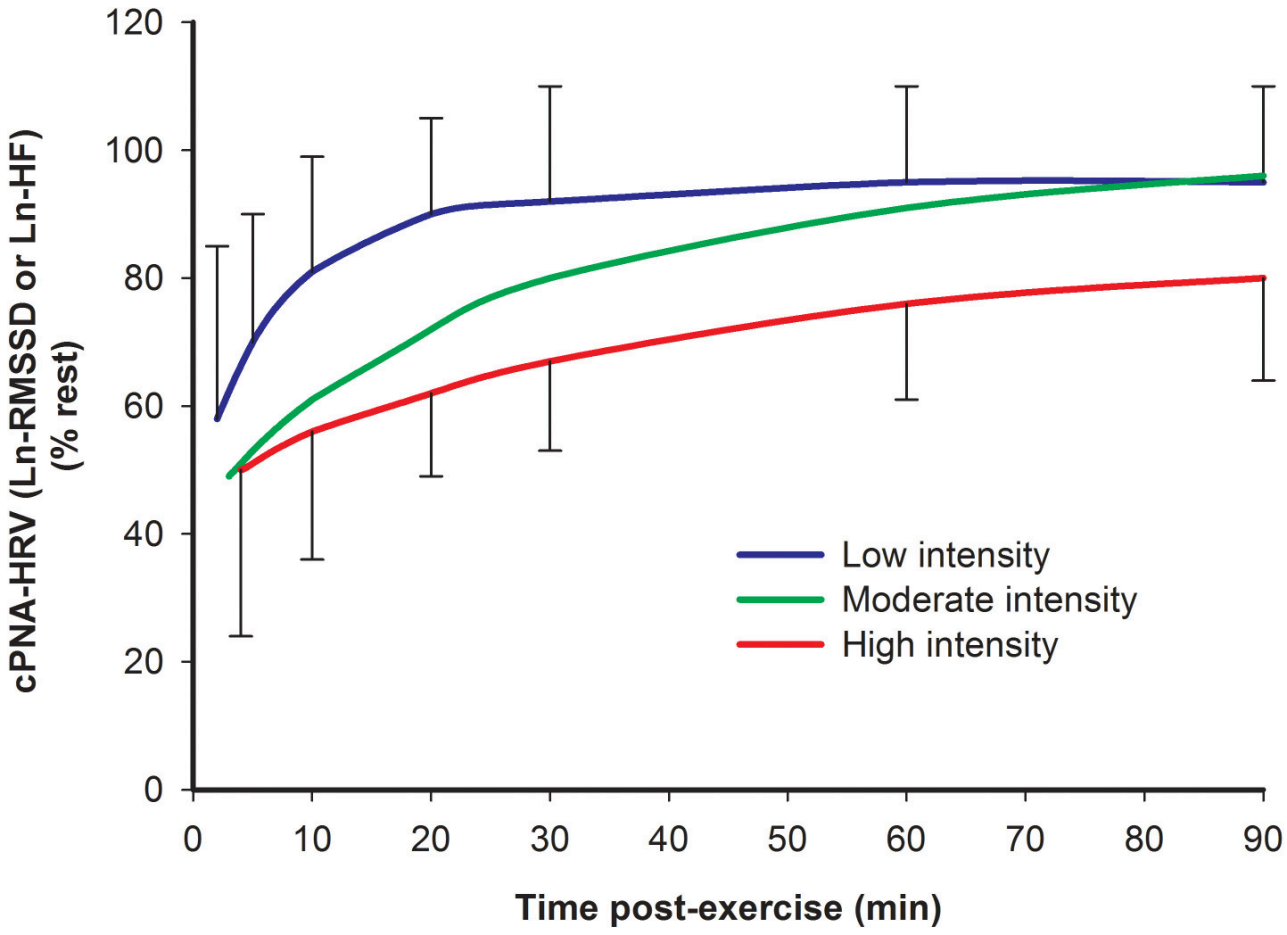
**Time course of cPNA-HRV recovery following different intensities of preceding exercise**. Ln natural log transform. RMSSD root mean square of successive differences. HF High frequency power. Low intensity: <70% VO_2_max. Moderate intensity: 70–82% VO_2_max. High intensity: >82% VO_2_max. Data are mean ± SD. Redrawn from Stanley et al. (2013).

Several studies have investigated HRV during recovery from different exercise intensities. A recent review (Stanley et al., 2013) quantitatively summarized the findings of some of these studies demonstrating that a higher exercise intensity is associated with a slower recovery of cPNA-HRV measures, specifically Ln-transformed RMSSD or HF (Furlan et al., 1993; Terziotti et al., 2001; Mourot et al., 2004; Parekh and Lee, 2005; Niewiadomski et al., 2007; Seiler et al., 2007; Kaikkonen et al., 2008). These data are redrawn in Figure 4. Other studies have reported overall similar findings for cPNA-HRV and other HRV measures, namely that a greater exercise intensity results in a slower HR and HRV recovery (Perini et al., 1990; Hayashi et al., 1992; Buchheit et al., 2007a; Kaikkonen et al., 2007, 2010, 2012; Martinmaki and Rusko, 2008; Gladwell et al., 2010; Al Haddad et al., 2011; Casonatto et al., 2011; Dupuy et al., 2012). From a mechanistic standpoint, the effect of intensity on HRV recovery is likely associated with the amount of non-oxidative energy contribution and subsequent stimulation of the muscle metaboreflex (Buchheit et al., 2007a).

However, the details of the intensity dose-response has not been clearly elucidated, particularly during the initial 10 min following exercise when comparing moderate vs. high exercise intensities. Most studies investigating HRV following more than two intensities report differences between some, but not all exercise intensities (Kaikkonen et al., 2007; Seiler et al., 2007; Gladwell et al., 2010; Casonatto et al., 2011). Seiler et al. (2007) suggested that the first ventilation threshold might demarcate an autonomic “binary threshold” regarding HRV recovery, whereby exercise below this intensity is associated with rapid HRV recovery while exercise above this intensity results in delayed HRV recovery which is intensity-independent (at least in highly trained athletes). In contrast, inspection of Figure 4 suggests that exercise intensity may elicit a more graded (rather than binary) effect on HRV recovery (at least during the first hour post-exercise). This latter interpretation is consistent with the findings of a recent study by this research group demonstrating a graded response following three different exercise intensities (Michael et al., 2016).

### Effect of exercise duration

Similar to responses during acute exercise, few studies have investigated the effect of exercise duration on HRV during recovery after exercise, in particular when controlling for the intensity. Seiler et al. (2007) investigated the recovery of HRV following either 60 or 120 min of low intensity running exercise (below VT1 at ~60% VO_2_max) in a highly trained athletic population. By the earliest time-point investigated post-exercise (5–10 min), HRV (including RMSSD) had recovered to pre-exercise levels and there was no significant effect of duration on HRV measures throughout the 4 h recovery period. These findings are consistent with those reported by Casonatto et al. (2011), who investigated the effect of exercise duration on HRV during 60 min of recovery by having healthy participants cycle for 30 min or ~45 min at 60% VO_2_max. There was no significant duration effect for the HRV measures assessed during recovery (RMSSD, LF-nu, HF-nu, and LF:HF).

The findings of Kaikkonen et al. (2007) also suggest a lack of any duration effect on HRV during the immediate recovery period. In that study, the time-course of immediate HRV recovery in sedentary women was investigated using STFT frequency-domain analysis. The participants performed low intensity (~45% VO_2_max) and moderate-high intensity (~77% VO_2_max) running for ~38 vs. ~76 min and ~30 vs. ~60 min for low and moderate-high intensity, respectively. Despite a strong effect of exercise intensity on the time-course of HRV recovery from exercise, there was no significant effect of exercise duration for either intensity from 1 to 30 min recovery.

While these studies suggest that a 100% increase in exercise duration does not alter HRV recovery following exercise, the results of another study by Kaikkonen et al. (2010) indicate that exercise duration may influence post-exercise HRV when duration is extended by 300–400%. In that study, recreation-level athletes ran at ~66% VO_2_max for ~20 and ~90 min. During the initial 3 min of a 15 min recovery, the longer duration resulted in lower Ln-LF, Ln-HF, and Ln-TP.

### Effect of exercise modality

Very few studies have examined post-exercise HRV recovery following different modalities of “aerobic” exercise. Cunha et al. (2015) investigated the immediate recovery period following incremental exercise of three modalities: walking, cycling, and running. During the 5 min recovery period, it was reported that HR recovery as well as the recovery of RMSSD was more rapid following exercise involving a smaller muscle mass or energy expenditure (i.e., cycling > walking > running), with the authors therefore concluding that muscle mass and/or energy expenditure are determinants of post-exercise parasympathetic reactivation. Similarly, active children demonstrated more rapid HR and HRV recovery following maximal incremental arm-cranking vs. cycling (Ahmadian et al., 2015), The findings of these two studies were also consistent with those of other studies investigating HR recovery (without HRV measurement) following exercise mode comparisons such as cycling vs. running (Rahimi et al., 2006; Maeder et al., 2009) and cycling vs. arm-cranking (Ranadive et al., 2011). These studies also utilized maximal incremental exercise. To our knowledge, HRV recovery has not been investigated following different exercise modalities utilizing submaximal intensity.

### Reliability of HRV during post-exercise recovery

During acute post-exercise recovery, the few studies investigating test-retest reliability have reporting conflicting results. Some studies report moderate to good relative reliability, such as ICC-values of 0.58–0.91 during active recovery (Boullosa et al., 2014) and 0.69–0.92 during static recovery (Carrasco et al., 2003) for various HRV measures. Alternatively, Dupuy et al. (2012) reported ICC-values ranging from 0.14 to 0.97 for during static recovery. Absolute reliability has also been reported to vary markedly, with TEM(CV) reported as 19–27% (Boullosa et al., 2014) and 8–65% (Al Haddad et al., 2011), while Dupuy et al. (2012) reported CVs ranging from of 27 to 141%. Considering the methodological differences amongst these few studies, it is difficult to draw any conclusions regarding the test-retest reliability of HRV during recovery.

### Summary—HRV during post-exercise recovery

A large body of research indicates that, as is the case during exercise, exercise intensity is a primary determinant of the immediate post-exercise recovery of HRV. Upon exercise cessation, HRV measures demonstrate a time-dependent recovery that is usually (though not always) slowed following a greater preceding exercise intensity. Thus, the intensity-dose response on HRV during recovery has not been clearly elucidated.

It is also not yet clear how exercise duration affects HRV during post-exercise recovery. While three studies report that a 100% increase in exercise duration does not alter HRV during immediate recovery, one study found that HRV recovery was slowed following a 300–400% increase in exercise duration (from ~20 to ~90 min). This may suggest that exercise duration must be prolonged beyond some critical length (either relative or absolute) before an effect on HRV recovery might be observed, however this remains speculative. Additionally, it is not clear how preceding exercise intensity and duration may interact to influence post-exercise HRV recovery. Finally, the two studies identified that investigated the effect of exercise modality on post-exercise HRV suggest that a greater active muscle mass and/or energy expenditure is associated with a slower HRV recovery, at least following maximal incremental exercise. Clearly, additional studies are needed to further elucidate the influences of exercise duration and modality on post-exercise HRV responses.

## Systolic time intervals

Although some measures of HRV (namely cPNA-HRV measures, i.e., RMSSD, HF, and SD1) are generally accepted to provide insight into cardiac parasympathetic modulation, HRV is not widely considered to reflect cardiac sympathetic activity (notwithstanding the controversial use of LF, LF-nu, and particularly LF:HF as indicators of sympathetic activity or “sympatho-vagal balance”). This represents an important gap in the measurement of cardiac autonomic activity, in particular because; (a) sympathetic hyperactivity is associated with increased risk of morbidity/mortality (Leenen, 1999; Mancia et al., 1999; Licht et al., 2010; Schwartz and De Ferrari, 2011; Shanks and Herring, 2013; Vink et al., 2013), and (b) as highlighted previously, there exists complex cardiac sympathetic-parasympathetic interactions, such that it may be difficult to interpret measures of one arm of autonomic activity in the absence of any information of the activity of the other arm. Systolic time intervals (STI) are another class of non-invasive measures that may provide valuable insight into cardiac sympathetic activity to complement cPNA-HRV measures.

### Physiological interpretation of STI

STI outcomes—such as the pre-ejection period (PEP), left ventricular ejection time (LVET), and PEP-to-LVET ratio (PEP:LVET)—are measures of cardiac performance that may be non-invasively measured using techniques such as bio-impedance cardiography. In particular, PEP may be used to assess cardiac sympathetic β-adrenergic activity (Ahmed et al., 1972; Sherwood et al., 1990; Cacioppo et al., 1994). The physiological rationale derives from sympathetic activity eliciting positive inotropic effects on the ventricular myocardium, thus increasing cardiac contractility and resulting in a more rapid development of force and intraventricular pressure. This decreases the time required to reach aortic pressure (the isovolumetric contraction time, IVCT) and therefore opening of the aortic valve, thereby attenuating PEP (hence PEP is inversely associated with cardiac sympathetic activity). In contrast to being richly innervated by cardiac sympathetic neurons, the ventricles are not extensively innervated by parasympathetic neurons, meaning that changes in contractility indices such as PEP are largely attributed to changes in cardiac sympathetic activity. Indeed, sympathetic stimulation by β-adrenergic agonists shortens PEP (Harris et al., 1967; Ahmed et al., 1972; Schachinger et al., 2001), whereas this response is reduced or abolished under conditions of β-adrenergic blockade (Harris et al., 1967; Benschop et al., 1994; Cacioppo et al., 1994). PEP:LVET has also been utilized as a measure of cardiac contractility, however LVET and PEP:LVET are influenced by the underlying HR (Cokkinos et al., 1976; Cacioppo et al., 1994) as well as parasympathetic activity (Cacioppo et al., 1994). In contrast, PEP is not substantially altered by changes in HR (Harris et al., 1967; Cokkinos et al., 1976). Thus, while it is widely acknowledged that LVET and PEP:LVET should be corrected for the underlying HR, the need for HR-correction of PEP (Weissler et al., 1968; Lewis et al., 1982) is contentious (Cokkinos et al., 1976; Spodick et al., 1984; Rousson et al., 1987; Cacioppo et al., 1994). As a result, PEP appears to be the STI measure of choice for reflecting cardiac sympathetic β-adrenergic activity (Rousson et al., 1987; Cacioppo et al., 1994).

The interpretation of PEP as reflecting cardiac sympathetic activity is not without limitations. In particular, non-sympathetic influences which may alter PEP need to be appreciated. Although the parasympathetic influence on the ventricles is considered to be weak, it is possible that parasympathetic outflow may inhibit positive inotropic sympathetic effects when sympathetic outflow is high (Levy, 1971; Azevedo and Parker, 1999; Figure 1). Another confounding factor relates to the fact that PEP encompasses the electromechanical delay as well as the IVCT. However, ~75% of the shortening of PEP in response to β-adrenergic agonists is due to shorting of IVCT (Harris et al., 1967), while changes during exercise and recovery are almost entirely accounted for by IVCT (Nandi and Spodick, 1977). In addition to these factors, cardiac loading may elicit non-sympathetic influences in PEP, in particular during exercise and post-exercise recovery. Namely, the Frank-Starling mechanism (length-dependent increase in cardiac contractile force, i.e., preload) and/or a decrease in aortic pressure (afterload) may shorten PEP (Lewis et al., 1977, 1982; Buch et al., 1980; Joubert and Belz, 1987). However, the extent to which preload and afterload actually change during exercise and recovery is debated (Plotnick et al., 1986; Kimball et al., 1993; Warburton et al., 2002; Rowland, 2008; La Gerche and Gewillig, 2010), as is the extent to which PEP reflecting cardiac sympathetic influences may be confounded (Nandi and Spodick, 1977; Cousineau et al., 1978; Lewis et al., 1982; Obrist et al., 1987). Future research is needed to clarify the extent to which these potential confounding factors influence PEP during exercise and recovery.

There are also methodological issues to consider. For example, bio-impedance cardiography is highly sensitive to movement artifact, which can make reliable signal acquisition difficult during exercise involving substantial upper body movement. Furthermore, postural changes as well as large and rapid thoracic movements (e.g., heavy breathing during hard exercise) can also influence thoracic impedance and make waveform identification and interpretation difficult.

### STI during exercise and recovery

The response of PEP to exercise is generally consistent with what would be expected of a true (inverse) cardiac sympathetic indicator, i.e., an intensity-dependent decrease from rest to exercise (Ahmed et al., 1972; Nandi and Spodick, 1977; Miyamoto et al., 1983; Miles et al., 1984; Smith et al., 1989). PEP has rarely been investigated during post-exercise recovery. Nandi and Spodick (1977) demonstrated that the gradual recovery of PEP during the initial 5 min post-exercise period is intensity dependent across a range of absolute submaximal intensities (50, 100, and 150 W), i.e., greater intensity elicited a slower recovery. PEP:LVET has also been reported to be lower following maximal (compared with submaximal) exercise (Crisafulli et al., 2006b), but not following different submaximal exercise intensities (Nandi and Spodick, 1977; Crisafulli et al., 2006b). Regardless, the response of PEP during exercise is consistent with our understanding of intensity-dependent sympathetic activation. Very limited data suggests that post-exercise PEP recovery is also consistent with intensity-dependent sympathetic withdrawal.

No study has investigated the effect of exercise duration on STI outcomes either during exercise or post-exercise recovery. Regarding the effect of exercise modality, inspection of the data reported by Miles et al. (1984) suggests that PEP may be lower during arm-crank exercise compared with cycling for a HR range of ~80 to ~150 b.min^−1^, however this is not clear due to different exercising heart rates. Alterations in posture (e.g., supine vs. upright cycling) would be expected to alter cardiac loading (in particular preload). Consistent with this, PEP during supine (compared with upright) cycling was reported to be lower at the same absolute intensities of 50 and 100 W, despite a lower HR at 100 W (Miyamoto et al., 1983), although a lack of any significant effect of posture on PEP during exercise has also been reported (Smith et al., 1989). Additionally, Crisafulli et al. (2006a, 2008) demonstrated that post-exercise muscle occlusion elicits an intensity-dependent delay in STI recovery. As for cPNA-HRV measures of post-exercise parasympathetic reactivation (see previous section), these findings highlight the important role that the muscle metaboreflex likely plays in regulating the post-exercise recovery of STI measures reflecting sympathetic withdrawal.

### Summary—systolic time intervals

STI measures (in particular PEP) are a useful non-invasive indicator of cardiac sympathetic activity. As is the case for HRV, STI measures reflect the integrated end-organ response, in this case indirectly assessing inotropic cardiac effects that are understood to be under strong sympathetic influence. Accordingly, interpretative caveats/limitations relating to non-sympathetic influences (particularly potential cardiac loading effects, i.e., preload and afterload) need to be appreciated. Notwithstanding these limitations, the measurement of PEP likely provides valuable non-invasive insights into cardiac autonomic regulation, as PEP is strongly (inversely) associated with cardiac sympathetic activity.

The effect of different exercise dosages on the response of PEP (or other STI outcomes) during exercise has not been extensively investigated. Nevertheless, available data indicates that the (inverse) response of PEP is consistent with our understanding of sympathetic activity during exercise and recovery. Additional studies are needed to confirm this, particularly during the recovery period. It is not known how exercise duration influences PEP responses to exercise and recovery, nor is it well established how these responses are altered by different exercise modalities. Additionally, the test-retest reliability of these measures during and following exercise requires further research.

## Overall summary and future perspectives

HRV is used as a non-invasive tool to monitor cardiac autonomic activity, with a wide range of quantitative approaches utilized. In particular, a large body of evidence indicates that cPNA-HRV measures (e.g., RMSSD, SD1, and HF) generally reflect cardiac parasympathetic modulation, although not without limitations. In contrast, the majority of evidence does not support the interpretation of HRV measures (in particular LF, LF-nu, and LF:HF) as reflecting sympathetic activity or “sympatho-vagal balance.”

Exercise elicits substantial changes in HRV measures, and several studies have investigated HRV during exercise and immediate post-exercise recovery. As is the case for studies investigating resting HRV measures, HRV analysis methodology varies widely. Regarding exercise and recovery, this issue is compounded by the wide range of exercise and recovery protocols employed. When combining these factors, very few (if any) studies investigating HRV during exercise and/or recovery are directly comparable, particularly when frequency-domain HRV measures are utilized. Additionally, some caution is advised when interpreting HRV measures calculated using very short epochs (e.g., 30 s) during non-stationary conditions (particularly during immediate post-exercise recovery), as non-oscillatory changes in HR may contribute to HRV. Nevertheless, a review of the literature concerning the effects of the primary exercise dosage factors for dynamic exercise (intensity, duration, and modality) reveals some noteworthy responses.

The literature indicates that the intensity of exercise is the primary exercise dose factor determining HRV responses during both exercise and post-exercise recovery. Most HRV measures demonstrate a reduction upon the initiation of exercise as well as an intensity-dependent decay toward near-zero levels. The intensity at which a near-zero minimum occurs depends upon the type of HRV outcome with cPNA-HRV measures typically reaching a minimum at 50–60% VO_2_max or around the first ventilation threshold. Normalized and ratio frequency-domain measures demonstrate inconsistent response during exercise that are not consistent with known aspects of cardiac autonomic activity. Upon exercise cessation, HRV recovers as a function of time, with a higher exercise intensity often (though not always) associated with a delayed recovery profile. Regarding the reliability of HRV during exercise and recovery, the few studies to date that have assessed test-retest reliability have reported mixed findings.

There is limited data on the effects of duration or modality on HRV during exercise and recovery. Methodologically, such investigations are difficult because of the close relationship between HR and HRV. Prolonged exercise duration has been associated with decreased HRV during exercise only when accompanied by a concomitant rise in HR. Prolonged exercise duration (100% increase) has been shown to elicit no significant influence on post-exercise HRV recovery, although a delayed recovery was reported when duration was prolonged by over 300% (e.g., from ~20 to ~90 min). Regarding modality, some studies report that the mode of exercise can alter the HRV response (even when HR is matched), although other studies have reported no significant modality effect. During recovery, very limited research suggests that modalities utilizing greater active muscle mass (and/or eliciting greater energy expenditure) are associated with slower HRV recovery, although this is preliminary. Potential intensity-duration and intensity-modality interactions on HRV during exercise have not been elucidated. Further research is required before conclusions on the effect of exercise duration and modality on HRV during exercise and recovery can be made.

Additional research is also needed regarding the effect of exercise dosage on non-linear HRV measures. Considering that HR is non-stationary during key segments of exercise and recovery, non-linear measures may be particularly useful for investigating HRV during these periods. Furthermore, the monitoring (prognostic/diagnostic) value of these measures requires further investigation.

Since HRV measures are not widely accepted to reflect cardiac sympathetic activity, it is noteworthy that STI indices likely provide useful non-invasive insights into cardiac performance. In particular, PEP is a valid (inverse) measure of cardiac sympathetic activity, notwithstanding some interpretative limitations. While a limited body of research indicates that the response of PEP to exercise and recovery is generally consistent with our understanding of cardiac sympathetic influences, the effects of different exercise dosages is not clear, particularly during the recovery period. Furthermore, the test-retest reliability of STI responses to exercise has not been established.

Finally, while HRV and STI reflect different aspects of cardiac function (chronotropic vs. inotropic), STI measures of cardiac sympathetic activity (PEP) may be used to complement HRV measures of cardiac parasympathetic activity (RMSSD, HF, and SD1) to provide a more comprehensive insight into cardiac autonomic regulation. Previous investigations have utilized this integrative approach under conditions of rest and psychological stressors (Berntson et al., 1994, 2008; Brindle et al., 2014). However, concurrent monitoring of the exercise stress-reactivity of HRV indices for cPNA and STI indices for cSNA during exercise and post-exercise recovery has not been reported. This integrative approach warrants further investigation.

## Author contributions

SM was the primary author who was responsible for the design and writing of the manuscript. KG and GD assisted with writing and editing the manuscript.

### Conflict of interest statement

The authors declare that the research was conducted in the absence of any commercial or financial relationships that could be construed as a potential conflict of interest.
